# Distinct temporal developments of visual motion and position representations for multi-stream visuomotor coordination

**DOI:** 10.1038/s41598-019-48535-0

**Published:** 2019-08-20

**Authors:** Hiroshi Ueda, Naotoshi Abekawa, Sho Ito, Hiroaki Gomi

**Affiliations:** NTT Communication Science Laboratories, Nippon Telegraph and Telephone Co., Kanagawa, Japan

**Keywords:** Sensorimotor processing, Motion detection, Motor control

## Abstract

A fundamental but controversial question in information coding of moving visual target is which of ‘motion’ or ‘position’ signal is employed in the brain for producing quick motor reactions. Prevailing theory assumed that visually guided reaching is driven always via target position representation influenced by various motion signals (e.g., target texture and surroundings). To rigorously examine this theory, we manipulated the nature of the influence of internal texture motion on the position representation of the target in reaching correction tasks. By focusing on the difference in illusory position shift of targets with the soft- and hard-edges, we succeeded in extracting the temporal development of an indirect effect only ascribed to changes in position representation. Our data revealed that the onset of indirect effect is significantly slower than the adjustment onset itself. This evidence indicates multi-stream processing in visuomotor control: fast and direct contribution of visual motion for quick action initiation, and relatively slow contribution of position representation updated by relevant motion signals for continuous action regulation. The distinctive visuomotor mechanism would be crucial in successfully interacting with time-varying environments in the real world.

## Introduction

To prevent a cup from falling off a tilted tray, we can quickly adjust our hand in response to sudden position change of the cup. How can we solve such complex visuomotor control problems in a very limited time with an object’s position and motion information, which would be signaled by separate pathways^1^ in the brain? To answer this question, we need to reveal the precise temporal dynamics of information processing representing position and motion, which is, however, still difficult to be assessed directly by brain imaging or neural recording techniques. One way to solve this is to tackle an important and controversial question whether the quick visually-evoked adjustments in reaching a target are directly driven by the target position information^2–5^. We intuitively consider that the planning and execution of such goal-directed reaching movements always require the position representation of the target. Interestingly, even a target is presented independently of its inside, background, or surrounding motion, the perceived target position is illusorily shifted in the direction of the visual motion: i.e., motion-induced position shift (MIPS)^4,6–11^. Several recent studies have shown that actions (manual pointing and saccadic eye movements), as well as perception, are biased by motion signals^12–17^. The prevailing view, therefore, assumes that quick manual reactions are induced, not directly by the visual motion itself, but indirectly by the illusorily shifted target representation regardless of whether or not we are perceptually aware of it^18^. On the other hand, because rapid responses are induced only by the background motion even in the absence of an explicit target^2^, a contrasting view predicts that visual motion can directly affect manual responses, not via influences on the representation of a target position^2,5^.

Here we dissociate the signals from direct and indirect pathways linking visual motion to manual control, based on their respective latencies. Specifically, we show that the motion-position interaction effect (i.e., the MIPS effect) on the reaching movement appears long *after* the initiation of rapid visuomotor responses and these rapid visuomotor responses are triggered directly by motion information.

## Results

**Figure 1:**
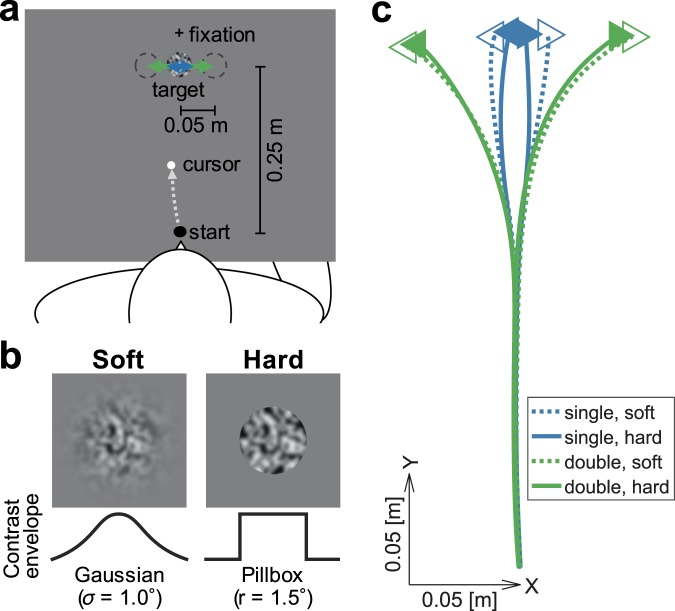
Experimental setup, stimuli, and hand trajectories. (**a**) Configuration of the experimental setup. Participants performed a reaching task while holding the handle of a manipulandum from the start position to the target position. In randomly selected trials, immediately after the reaching onset, the internal texture of the target stimulus started to move in the horizontal direction with or without the edge position drifting (single- and double-drift conditions, respectively). Participants quickly made an online adjustment when the target position shifted. (**b**) Target stimuli. The soft- and hard-edge targets were created by applying the contrast envelope of a Gaussian function and a pillbox function, respectively, to a Gaussian smoothed random dot pattern. (**c**) Mean trajectories for single-drift (blue) and double-drift (green) conditions of a particular participant. The dotted curves and open triangles represent the trajectories and arrival positions for the soft-edge target, and the solid curves and filled triangles represent those of the hard-edge target. The direction of the triangle indicates the direction of the texture motion.

In the experiment, we adopted an online reaching task (Fig. 1a). The internal texture started to drift in a horizontal direction with or without an accompanying target shift in the same direction (double- and single-drift conditions, respectively). Two other target conditions were also used to prevent anticipatory responses (see Methods for details).

To examine the temporal development of the MIPS effect on adjustments in reaching, we focused on a difference in the strength of MIPSs induced by two kinds of targets. A soft-edge target (Fig. 1b, left panel) presented in the peripheral vision is known to cause a large MIPS for perception and action while a hard-edge target (Fig. 1b, right panel) induced a significantly small and negligible MIPS for perception and action, respectively^13^. The effect of edge difference has been successfully modeled by a Kalman filter, which optimally weights the motion and position signals based on their certainty^19^ (see also^20^). Therefore, based on these studies, if rapid adjustments in reaching evoked by visual motion are mediated by changes in target position representations, a stronger response to the soft-edged, compared to the hard-edged, target should be observed from the very beginning.

**Figure 2:**
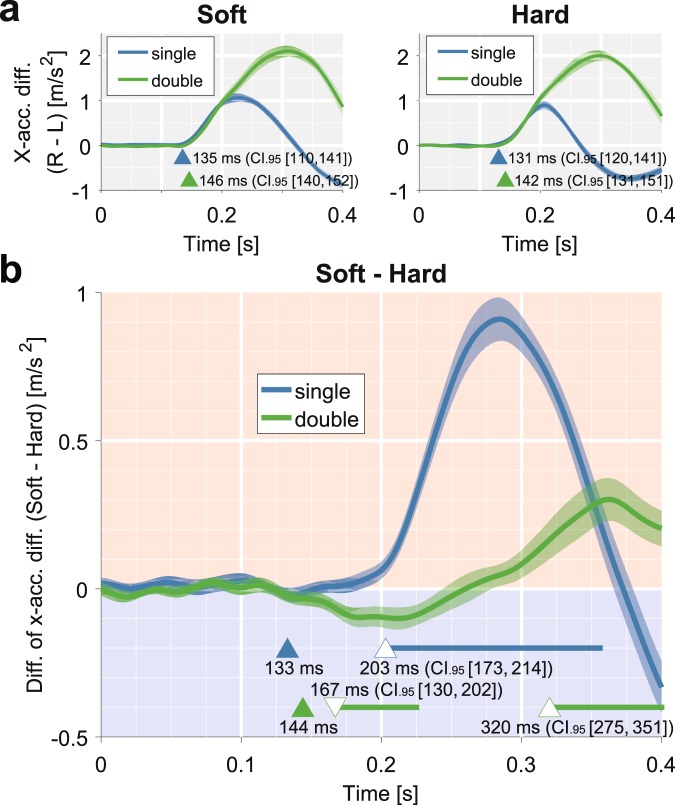
Mean adjustment responses (horizontal acceleration of the hand) of all participants (*N* = 20). (**a**) Responses for the soft-edge target (left panel) and the hard-edge target (right panel). The responses to the single- and double-drift targets are shown with blue and green lines, respectively (*M* ± *SEM*). The time zero denotes the onset of the target motion. The triangles indicate the adjustment response onset (*p* < 0.01 with one-tailed successive *t*-tests). (**b**) The differences between the correcting responses of the soft- and hard-edge targets under double-drift (green curve) and single-drift (blue curve) conditions (*M* ± *SEM*). The positive deflection indicates the effect of MIPS. The filled triangles indicate the mean of the adjustment response onset times for the soft- and hard-edge targets. The bottom horizontal lines denote the periods during which the responses were significantly deflected from zero, and the open triangles indicate those onsets (*p* < 0.01 with one-tailed successive *t*-tests; the directions of the triangles indicate positive and negative deflection). The 95% confidence intervals (CIs) were obtained by bootstrapping across participants (see Methods and Supplementary Fig. 3).

Consistent with previous studies^13,19,21^, the shift of the final hand position was greater for a soft-edge target than for a hard-edge target in both single- and double-drift conditions (*t*(19) = 17.84, *p* < 0.001, and *t*(19) = 4.33, *p* < 0.001, respectively; e.g., Fig. 1c). To quantify the responses for each target, we first took the difference between the horizontal accelerations (rightward − leftward) of the hand movements (Fig. 2a). Manual responses for single- and double-drift conditions started with a short latency (<150 ms) and diverged at about 200 ms for both the soft- and hard-edge targets.

To highlight the temporal development of the MIPS effect, we subtracted the responses to the hard-edge targets from the responses to the soft-edge targets (Fig. 2b). Any time-windows showing a positive difference (the red area in Fig. 2b) indicate adjustments attributed to the MIPS effect. As mentioned above, if a fast manual response is triggered by a shift of the target position representation, the difference should become positive from the adjustment onset. However, as is evident from the blue line in Fig. 2b, the difference in the responses emerged (open triangle) approximately 70 ms *after* the onset of the motor adjustments (filled triangle). Therefore, the MIPS effect cannot induce the initial motor adjustment. One could argue that the slow increase of the MIPS effect can be ascribed to masking of the MIPS effect by the greater effect of the internal texture motion in the hard-edge target due to its higher luminance contrast used in the experiment. However, this slow increase was also confirmed in a control experiment by comparing soft- and mixed-edge targets with similar inside motion energies (Supplementary Fig. 1).

Interestingly, in the double-drift condition, the difference in the responses (the green curve in Fig. 2b) became *negative* as soon as the adjustment responses were initiated and then became positive approximately 180 ms later. The initial negative difference indicates that the motion energy of the hard edge itself is greater than that of the soft edge. In fact, in a further control experiment using a target with only a position shift (i.e., without internal texture motion), we found that the difference in the responses became negative around the response initiations (Supplementary Fig. 2). Following the initial negative deflection (green curve in Fig. 2b), the difference in the double-drift condition increased in the positive direction after 0.2 s. Consistent with our interpretation of the single-drift condition (blue curve), this late increase in the double-drift condition (green curve) can be attributed to the MIPS effect.

## Discussion

Here we characterized the temporal developments of the interaction effect between motion and position information, without direct effect of visual motion on reaching movement. A recent study^13^ showed that the MIPS effect influences the landing position of saccades with short-latency (100–175 ms), suggesting that the target-oriented motor response relies on the position representation, as in perception. However, the onset of the MIPS effect is difficult to be determined from the saccade landing positions since the MIPS processing may already be started before or during the saccades. Another study^18^ also estimated that the latency of the MIPS effect is approximately 120 ms based on the delay form the reversal of background motion to the change in the reaching trajectory, but even that experiment could not discriminate between the direct and indirect effects of background motion on the motor system^2,5^.

Here we have decoupled the initial manual adjustment to the target displacement into the quick direct-effect of the motion and the relatively slow indirect-effect caused by motion-position interaction. Most notably, the MIPS effect is slower than previously thought. This slow build-up of the MIPS effect is reasonable from the viewpoint of computational complexity since additional computation must be required for the integration of motion and position information^19^. This new evidence regarding the different time-courses of these two mechanisms sheds new light on the controversy concerning the effects of position and motion on quick manual adjustment. It also serves as an essential clue in revealing the internal representations of dynamic and multi-stream brain processing for stabilizing visuomotor control.

## Methods

### Participants

Twenty paid volunteers (seven men and thirteen women all right-handed, aged 21–50 years: *M* = 35.8, *SD* = 9.51) participated in the experiment. None had motor or visual disorders and all had normal or corrected-to-normal vision. They all gave written informed consent prior to participation. All experimental protocols were approved by the NTT Communication Science Laboratories Research Ethics Committee and were in accordance with the Declaration of Helsinki.

### Experimental setting and apparatus

Participants were seated and grasped the handle of a manipulandum (KINARM End-Point Lab, Bkin Technologies) with their right hand. The handle was free to move in the horizontal plane, and its position was recorded at a sampling rate of 1 kHz. All visual stimuli including a cursor (a white dot with a radius of 0.4 cm) representing the hand position were displayed on a horizontal mirror placed on the movement plane and reflecting the display of a 47-inch LCD monitor (47LS35A, LG) with a refresh rate of 60 Hz. The mirror prevented the participants from seeing their arm. A forehead rest was used to stabilize each participant’s head at a viewing distance of about 57 cm from the initial target position (40 cm in front). Participants’ gaze position was monitored with an infrared eye tracker (EyeLink 1000 remote, SR Research) to confirm that their eyes were on the fixation point during the trials.

### Stimuli and procedure

The moving targets (Fig. 1b) with a different contrast envelope to Gaussian-smoothed random-dot patterns (dot size = 0.16°: *σ* = 0.1°) were shown using MATLAB (MathWorks) and the Psychophysics Toolbox^22–24^. The contrast envelope of the stimulus was either a Gaussian function (*σ* = 1.0°) with an outer boundary at a diameter of 6.0° (a soft-edge target: RMS contrast = 11.82%) or a pillbox function with an outer boundary at a diameter of 3.0° (a hard-edge target: RMS contrast = 16.09%).

A schematic representation of the experimental setting is shown in Fig. 1a. Each trial started with the presentation of a randomly selected target stimulus (soft- or hard-edge) with equal probability on a gray background (12.2 cd/m^2^). The participants moved their hands to the start position (a black circle with a radius of 0.8 cm) located 25 cm in front of the target and directed their gaze at a fixation point (a 1.0 by 1.0 cm black cross with a width of 0.2 cm) located 5 cm ahead of the target. The start circle and the fixation cross were presented throughout the experiment. After a 200-ms delay, the target stimulus was presented at its initial position and a tone (1510 Hz for 150 ms) was sounded as a cue to start a reaching movement. Movement onset was defined as the time at which the hand position was 1 cm from the start position. The participants were required to move their hands as close as possible to the center of the (final) target position within a time window of 600 ± 150 ms. The target disappeared 400 ms after the target motion onset. Another tone was also sounded 600 ms after the movement onset as a cue for the movement time. The final hand position was defined as the position 100 ms after the hand had remained within the 3 by 6 cm region centered on the final target position.

In one-third of the trials, the target remained at its initial position without any internal texture motion (the control condition). In the remaining trials, the target exhibited either texture motion (single-drift), both texture and edge motions in the same direction (double-drift), or both texture and edge motions in different directions (incongruent double-drift), with equal probability. The control and incongruent double-drift conditions were introduced to prevent the participants from providing an anticipatory response and to prompt them to respond to position changes, and therefore, these data were not further analyzed. The speeds of the texture and edge motions were 18.75 cm/s and 12.50 cm/s, respectively. The directions of the texture and edge motions (rightward or leftward) were chosen randomly with equal probability. The target motion started when the hand position was 1 cm from the start position. The participants were required to make an online adjustment in reaching as quickly as possible when the target position shifted (in response to the edge motion but not to the texture motion).

Each participant completed ten blocks of 54 trials, of which the first block was treated as practice. Each block consisted of three repetitions of all combinations of edge type (soft and hard), motion condition (single-drift, double-drift, and incongruent double-drift), and motion type (rightward, leftward, and no-motion, where no-motion resulted in the same static target for all motion conditions). The stimulus presentation order was randomized for each block and participant. A trial was regarded as a failure if the movement time exceeded the 450–750 ms time window or the final cursor position was outside the 3 by 6 cm region. Failed trials were informed by five auditory tones after each trial and were repeated at a random time later within the same block.

### Data analysis

All kinematic data related to hand position, velocity, and acceleration were low-pass filtered by using a sixth-order, zero-lag Butterworth filter with a cutoff frequency of 20 Hz. Trials were discarded from the analysis if any of the kinematic data (400 ms from the movement onset), movement time, or the final hand position, had deviated by more than 3 times the interquartile range from the 25^th^ or 75^th^ percentile of the data of each condition (boxplot outlier identification procedures). Less than 10% of the trials of each participant were excluded based on this criterion.

For each participant, the temporal development of the adjustment responses of each edge type (soft and hard) and motion condition (texture and edge) were quantified by taking the difference between the mean horizontal accelerations of the manual response to the rightward and leftward targets (rightward − leftward). The latencies of the adjustment responses of each condition were defined as the time point at which the *p*-value of a one-tailed *t*-test on the mean adjustment responses first fell below 0.01 after 100 ms had passed since the target onset and remained below 0.01 for 20 ms. The difference between the onset of response of the soft- and hard-edge targets was also defined in the same way as the response onset; the time at which the *p*-value of a one-tailed paired *t*-test between the adjustment responses of the soft- and hard-edge targets first fell below 0.01 (after 100 ms had passed since the target onset) and remained at 0.01 for 20 ms. To determine both periods when the adjustment response to the soft-edge target was greater than that to the hard-edge target and vice versa, a one-tailed paired *t*-test was conducted in both directions.

### Statistics

To evaluate the statistical differences between the response latencies (defined by rightward vs. leftward target motion) and the response difference onsets (defined by soft- vs. hard-edge targets), we adopted a bootstrap confidence interval procedure. In particular, from the original data set of all the participants (*N* = 20), a bootstrapped data set with the same sample size was nonparametrically resampled with replacement. The response latencies and the response difference onsets were then obtained from the bootstrapped data set in the same way as that employed for the original data set. We repeated the same procedure 2000 times to verify whether or not the 95% confidence intervals (2.5^th^ to 97.5^th^ percentile) of the distribution of each onset time overlap. The bootstrap data sets whose response latency or response difference onset time was not obtained with our criteria (i.e., where the *p*-values of one-tailed successive *t*-tests did not fall below 0.01 for more than 20 ms between 100 and 400 ms after the target onset) were excluded from the distributions.

## Supplementary information

Supplementary Figures
