# Supplementary material for: Distinct temporal developments of visual motion and position representations for multi-stream visuomotor coordination
Source: Sci Rep. 2019 Aug 20;9:12104. doi: 10.1038/s41598-019-48535-0 (PMC6702162; doi:10.1038/s41598-019-48535-0)
Supplement: Supplementary file 1 — Supplementary Figures [file 41598_2019_48535_MOESM1_ESM.pdf]

Supplementary Figures

Distinct temporal developments of visual motion and position representations for multi-stream visuomotor coordination

Hiroshi Ueda[1]*, Naotoshi Abekawa[1], Sho Ito[1] & Hiroaki Gomi[1]*

[1]NTT Communication Science Laboratories, Nippon Telegraph and Telephone Co., Kanagawa, Japan

*E-mail: hiroshi.ueda.be@hco.ntt.co.jp, hiroaki.gomi.ga@hco.ntt.co.jp

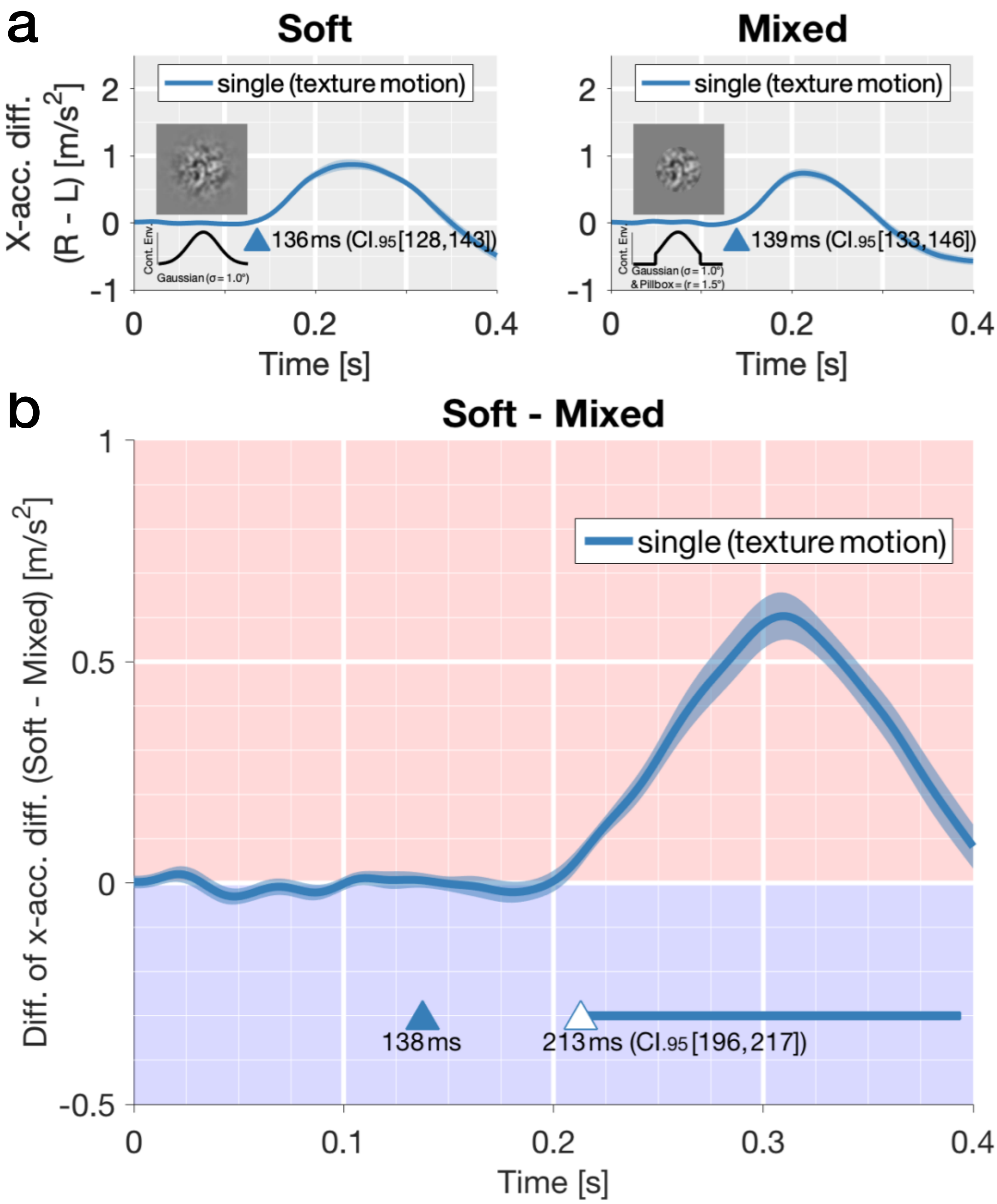

Supplementary Figure 1. Comparison of adjustment responses for soft- and mixed-edge targets in the single-drift condition.

(**a**) The mean adjustment responses (the difference between the hand accelerations for the rightward and leftward stimuli: rightward − leftward) for a soft-edge target (left panel) and a mixed-edge target (right panel) ($M \pm SEM$: N = 20). The contrast envelope of the soft-edge

target was a Gaussian function ($\sigma = 1.0°$) with an outer boundary at a diameter of 6.0° (i.e., the same as in the main experiment: RMS contrast = 11.58%) and that of the mixed-edge target was the same Gaussian function ($\sigma = 1.0°$) with a hard boundary at a diameter of 3.0° (RMS contrast = 10.91%). The speed of the texture and edge motions were 12.50 cm/s. The experimental procedure was the same as that used in the main experiment. The times at which the responses to rightward and leftward targets significantly diverged (i.e., adjustment response onset: $p < .01$ by one-tailed successive *t*-tests) are indicated by the triangles. The x-axis denotes the time from the onset of the target motion. (**b**) The differences in the correcting responses between the soft-edge target (**a**, left panel) and the mixed-edge target (**a**, right panel) in the single-drift condition (*M* ± *SEM*). The positive deflection of the difference indicates the effect of MIPS. The filled triangle indicates the mean adjustment response onset time for the soft- and mixed-edge targets (138 ms). The horizontal line beneath the plot indicates the time period during which the responses for the soft- and mixed-edge targets were significantly different ($p < .01$ by one-tailed successive *t*-tests), and the open triangles indicate the onset (213 ms: the direction of the triangles indicates the direction of the one-tailed *t*-test). Therefore, as in the main experiment, it was confirmed that the MIPS effect caused by the soft-edge target lags approximately 70 ms after the initial response even when compared with a stimulus (the mixed-edge target) with a weaker inside motion signal.

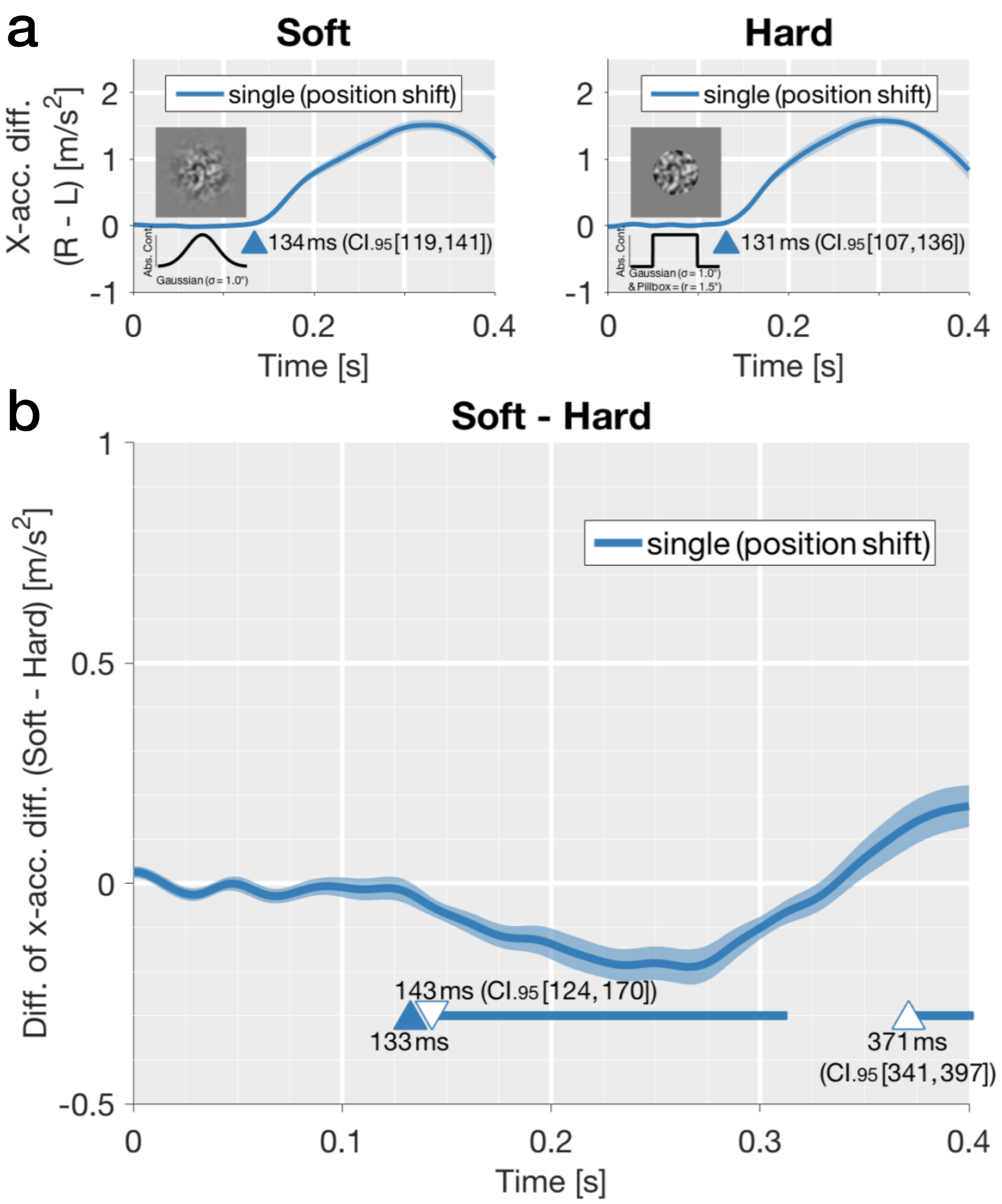

Supplementary Figure 2. Comparison of adjustment responses for the positional drifts (no internal texture motion) of soft- and hard-edge targets.

(**a**) The mean adjustment responses (the difference between the accelerations for the rightward and leftward stimuli: rightward − leftward) for a soft-edge target (**a**, left panel) and a hard-edge target (**a**, right panel) ($M \pm SEM$: $N = 20$). The contrast envelopes of the soft-edge target (RMS

contrast = 11.58%) and of the hard-edge target (RMS contrast = 16.22%) were identical to those of the main experiment. The speed of the edge motion was 12.50 cm/s. No texture motion was applied. The experimental procedure and the data analysis process were the same as in the other experiments. The filled triangle in each panel indicates the time at which the response to rightward and leftward targets significantly diverged (p < .01 by one-tailed successive t-tests). (**b**) The differences between the responses of the soft-edge target (**a**, left panel) and the hard-edge target (**a**, right panel). The filled triangles indicate the mean response onset time for the soft- and hard-edge targets (133 ms). The horizontal line beneath the plot indicates the period during which the responses for the soft- and hard-edge targets were significantly different (p < .01 by one-tailed successive t-tests), and those onsets are denoted by the open triangles (371 ms for the positive and 143 ms for the negative directions, respectively; the direction of the triangles indicates the direction of the one-tailed t-test). Note that unlike other conditions, since there is no internal texture motion, the positive deflection of the difference does not mean the effect of MIPS. Instead, the positive and negative deflections indicate that larger responses were induced by shifting the soft- and hard-edge targets, respectively. Thus, the fact that the negative deflection occurred immediately after the mean response onset suggests that the hard-edge target motion contains a larger signal to trigger a manual response than the soft-edge target.

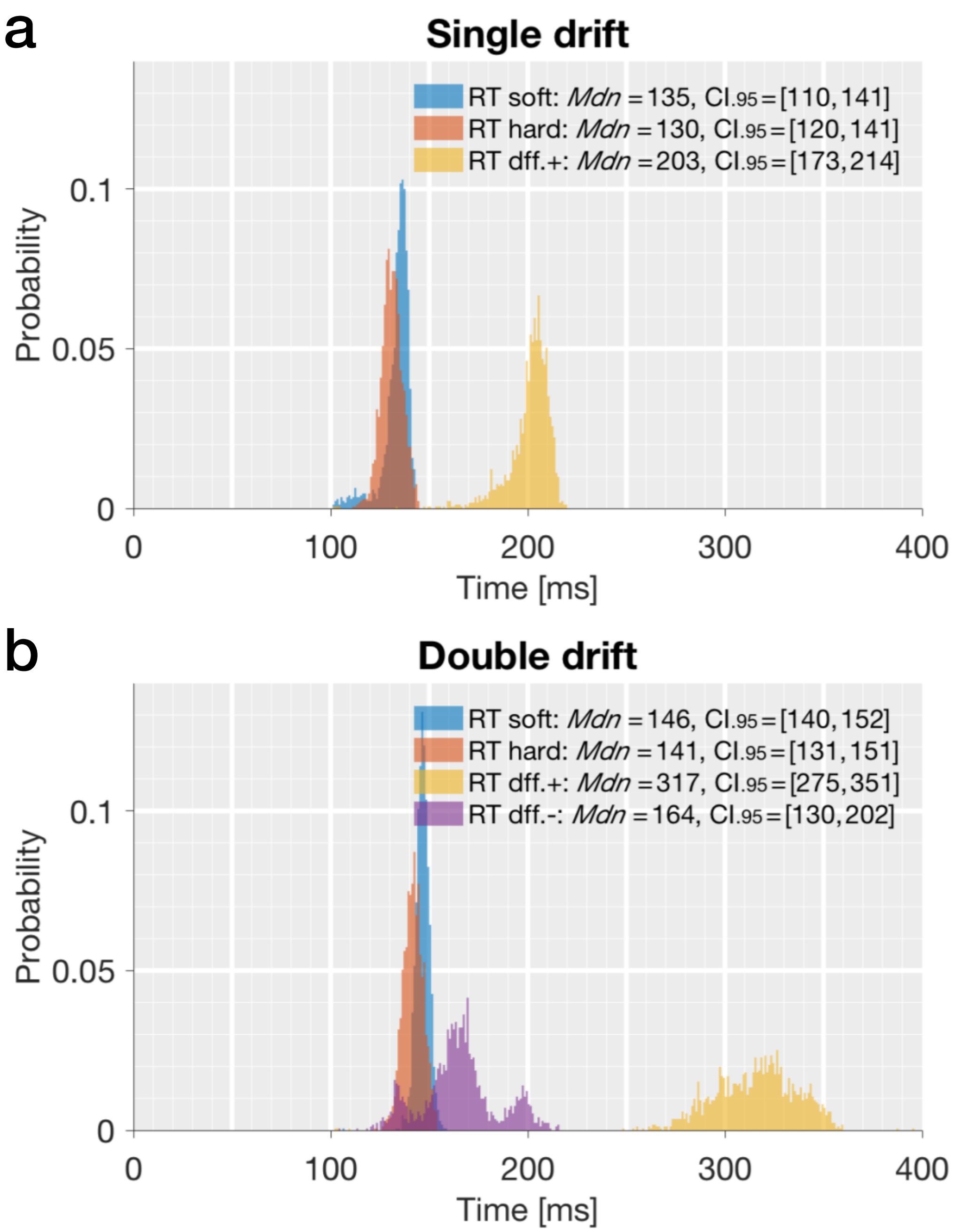

Supplementary Figure 3. Bootstrap distributions/histograms of response latencies and difference onset times (n = 2000) in (a) the single-drift condition and (b) the double-drift condition.

Each response latency and difference onset time were obtained from a bootstrapped data set that nonparametrically resampled from the original data set of all participants (N = 20) with

replacement. The criteria used to obtain the response latencies and difference onset times were identical to those applied to the original data set (see Methods for details). According to the 95% bootstrap confidence intervals (2.5th to 97.5th percentile), the onset of the MIPS effect (i.e., the positive difference in the response to the soft minus the hard target: yellow bars) lagged initial responses (blue and red bars) in both single- and double-drift conditions. On the other hand, the direct motion effect (i.e., the negative difference in the response to the soft minus the hard target: purple bars) occurred at a time comparable to that of the initial response in the double-drift condition.
